# Impact of ventilation and ambient temperature on COVID-19 transmission in clinic waiting rooms: A computational fluid dynamics approach

**DOI:** 10.1371/journal.pone.0328154

**Published:** 2025-08-08

**Authors:** Zhankun Zhu, Guosheng Gao, Yaoren Hu, Xiansheng Zhao

**Affiliations:** 1 General Affairs Department, Ningbo No.2 Hospital, Ningbo, China; 2 Department of Clinical Laboratory, Ningbo No.2 Hospital, Ningbo, China; 3 Department of Liver Disease, Ningbo No.2 Hospital, Ningbo, China; Wuhan University of Technology, CHINA

## Abstract

The ongoing COVID-19 pandemic underscores the necessity of understanding the transmission dynamics in enclosed, high-risk environments, such as clinic waiting rooms. This study used computational fluid dynamics (CFD) to investigate the behavior of virus-laden aerosols in clinic waiting rooms under six different scenarios with various temperatures and ventilation setups, offering insights into practical strategies for enhancing safety in healthcare environments. Key findings demonstrated that effective ventilation, through open windows and mechanical systems, can reduce virus-laden aerosol concentrations by up to 99.3% under optimal conditions (e.g., from 5.80 kg/m^3^ to 0.04 kg/m^3^By contrast, poorly ventilated scenarios exhibit significantly higher viral concentrations, which can rise as high as 5.80 kg/m^3^. A novel aspect of this research lies in the comprehensive modeling of human anatomy and aerosol interactions, which enhances the accuracy of viral-trajectory predictions. The practical implications include strategic recommendations for ventilation system design to mitigate transmission risks in clinical settings. These insights provide guidelines for healthcare facility design and emphasize the critical role of environmental control in reducing exposure to airborne pathogens.

## 1 Introduction

### 1.1 Context and background

Coronavirus disease (COVID-19) has emerged as a significant and persistent global health challenge since its outbreak in late 2019, infecting millions and causing widespread morbidity and mortality [1]. The coronavirus spreads primarily through respiratory droplets and aerosols, which are expelled when an infected individual coughs, talks, or breaths. These airborne particles can be inhaled by nearby individuals, triggering a cascade of infections [2]. The pathogenicity of the virus lies in its ability to bind to the ACE2 receptor, a protein widely distributed in the lungs, heart, kidneys, and intestines, thereby facilitating its entry into host cells [3]. This mechanism underpins the diverse clinical manifestations of the virus, ranging from asymptomatic infections to severe respiratory distress requiring mechanical ventilation, and in extreme cases, fatal outcomes [4].

The pandemic has necessitated rapid advancements in treatment and prevention strategies. Vaccination campaigns have emerged as the cornerstone of efforts aimed at controlling the spread of the virus [5]. However, the impact of the pandemic has been multifaceted, extending beyond healthcare to profoundly disrupt socioeconomic structures worldwide. Measures such as lockdowns and restrictions, which were adopted to curb the transmission of COVID-19, have placed immense pressure on global economies, with healthcare systems facing overwhelming demands. Additionally, the psychological toll of isolation and uncertainty has led to a surge in mental-health concerns, further complicating societal responses to the pandemic [6,7].

Treatment protocols for COVID-19 continue to evolve, including antiviral therapies, anti-inflammatory agents, and supportive care measures [8]. Despite these advances, significant challenges remain, particularly the emergence of new viral variants that exhibit increased transmissibility or resistance to existing interventions [9]. Asymptomatic carriers further complicate detection and containment efforts, highlighting the critical need for robust diagnostic tools, widespread testing, and effective contact tracing strategies to manage and mitigate the spread [10].

### 1.2 Computational fluid dynamics (CFD) in studying transmission dynamics

Clinic waiting rooms, characterized by a high occupant density, variable ventilation conditions, and prolonged exposure duration, represent environments with a high risk of transmission of respiratory infections [11]. A thorough understanding of aerosol-transmission dynamics is required to implement effective infection-control measures in these settings. CFD has emerged as a critical tool in this aspect, providing detailed insights into the flow and dispersion of aerosols and droplets within enclosed spaces [12].

CFD models simulate the interaction of fluids with surfaces and occupants, enabling the analysis of airflow, pressure, temperature, and species concentrations in various environmental scenarios [13]. Owing to these capabilities, CFD models can replicate real-world conditions in clinic waiting rooms by considering factors such as ventilation systems, room geometry, and occupant behavior. By visualizing potential transmission pathways, CFD helps evaluate and optimize mitigation strategies, particularly in high-risk healthcare settings [14,15].

Numerous studies have demonstrated the applicability of CFD in addressing the challenges posed by the airborne transmission of COVID-19. For instance, Saqr [16] highlighted the significance of reproducibility in CFD studies related to SARS-CoV-2 transmission, whereas Korhonen [17] investigated the use of MATLAB-based three-dimensional (3D) CFD analyses for modelling shear-driven indoor airflows and offered valuable insights into virus dispersion patterns. Similarly, Gulec et al. [18] employed a molecular communication framework to statistically characterize pathogen transmission in turbulent channels, providing novel perspectives on aerosol behavior. Pal et al. [19] extended this understanding by examining classroom transmission scenarios and proposed sustainable ventilation solutions tailored for such environments.

The influence of environmental factors on aerosol dynamics has also been extensively studied. Wang and Hong [20] investigated the risk of airborne transmission in low-ceiling rooms with mechanical displacement ventilation, whereas Park et al. [21] assessed the effects of ventilation strategies on airflow distribution and disinfection performance in various settings, including walk-through booths and classrooms. Kumar et al. [22] provided critical insights into the dispersion of sneeze-generated droplets in a meat-processing facility, highlighting the importance of spatial partitioning. Qiu et al. [23] used CFD to analyze the physiological impacts of COVID-19, identifying the alterations in airway resistance following the inception of the infection.

Several studies have emphasized the role of ventilation in mitigating transmission risks in healthcare facilities. Firatoglu [24] examined the effects of natural ventilation in classrooms, whereas Amahjour et al. [25] used CFD and Lagrangian coherent structures to analyze SARS-CoV-2 propagation in hospital isolation rooms. Dental clinics have also been investigated; Karami et al. [26] modeled the dispersion of aerosols generated from coughing to identify safe areas and appropriate ventilation velocities. Additionally, Dey et al. [27] quantified the effectiveness of various strategies employed to minimize aerosol dispersion in these high-risk environments.

Numerous studies have examined the broader implications of airflow management for infection control. Perić and Perić [28] demonstrated that even minor gaps in mask fit can substantially impair protection, allowing droplet escape. Sivakumar et al. [29] highlighted the energy-efficiency benefits of wind-catcher optimization for natural ventilation, whereas Tripathi and Moulic [30] analyzed the impact of outlet positioning on airflow patterns, providing actionable insights for medical-facility designs. Additionally, Armand and Tâche [31], Song et al. [32], Liu and Deng [33], and Luo et al. [34] have expanded the understanding of ventilation systems, particle transport, and the influence of architectural features on pathogen dispersion.

Studies on aerosol dynamics and droplet dispersion in confined spaces, such as elevators, have further underscored the complexity of mitigating airborne transmissions [35]. Computational frameworks such as those proposed by Vita et al. [36] and Tan et al. [37] continue to push the boundaries of predictive modeling, offering valuable tools for public-health planning and infection control.

### 1.3 Purpose and approach of this study

This study employed CFD to model the interactions between airflow, human anatomy, and virus-laden droplets in clinic waiting rooms, providing insights into the transmission dynamics of COVID-19. A key innovation is the incorporation of detailed human-body modeling and the discrete phase model (DPM) technology, which enables an accurate tracing of droplet trajectories and identification of high-risk zones for viral transmission. Six scenarios reflecting summer and winter conditions with various ventilation configurations were simulated to evaluate the effects of airflow and environmental factors on the droplet behavior. Although direct experimental validation was not feasible, the results were compared with those of previous studies, demonstrating an alignment with established aerosol dynamics. The findings of this study underscore the importance of effective ventilation as a simple yet powerful means to reduce transmission risks. By implementing the practical recommendations provided in this study, healthcare facilities can create safer environments for patients and medical staff, thereby fostering trust and wellbeing.

## 2 Methodology

### 2.1 System modeling

To unravel the intricacies of COVID-19 transmission in confined clinical environments, a CFD framework was established, which centered on a prototypical small clinic waiting room under both summer and winter conditions.

**Fig 1:**
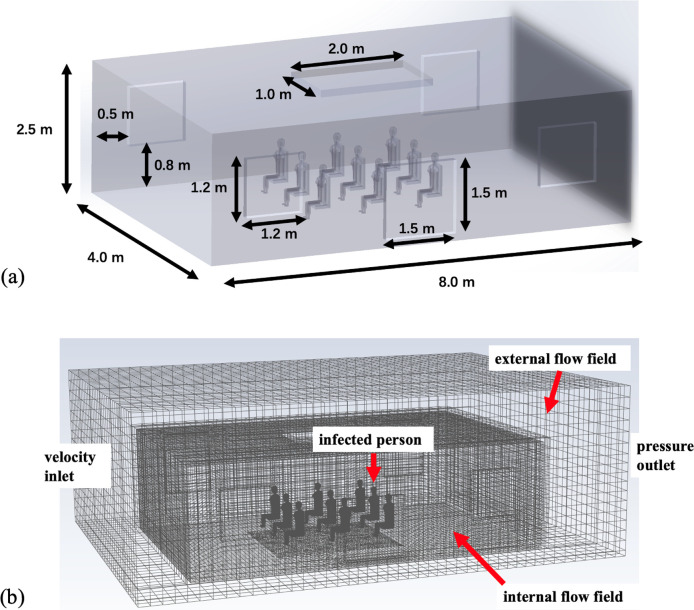
(a) Schematic layout of the clinic waiting room showing walls, windows, and centralized ventilation system. (b) Coupled computational domain illustrating concurrent outdoor wind and indoor airflow dynamics.

The virtual spatial design, illustrated in Fig 1(a), encompasses a clinic waiting room with dimensions of 8.0 m × 4.0 m × 2.50 m, which was approximately derived from the layout of an actual local clinic. Critical to the model is the inclusion of two small windows on both front and rear façades, each with dimensions of 1.2 m × 1.2 m. Additionally, a larger window with dimensions 1.5 m × 1.5 m is incorporated into the front wall, the position and size of which were extrapolated from the architecture of the local clinic through observations. An integral part of the model is the centralized ceiling ventilation apparatus, with dimensions 2.0 m × 1.0 m × 0.2 m, which aims to facilitate the active expulsion of indoor air to the external environment.

Fig 1(b) shows the synchronous modeling of both outdoor wind flow and indoor air circulation within a singular computational domain. A coupling method was adopted by linking the outdoor and indoor flow fields. The entire computational space measured 12 m × 8 m × 4 m. Notably, nine clinic attendees were seated in a 3 × 3 configuration. The model positioned an infected individual at the center of the posterior row, with a head height approximately equal to that of the attendees.

**Fig 2:**
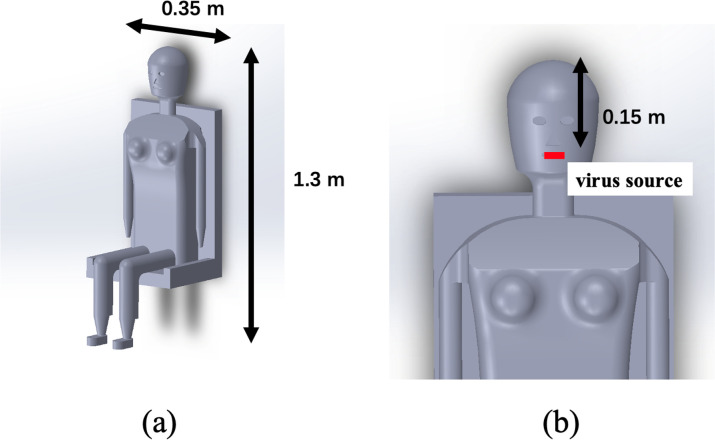
(a) Morphological representation of a seated human with specified dimensions. (b) Close-up view highlighting the aerosol emission zone from the infected patient’s mouth.

An intricate and detailed human body model was developed, with the seated human figure extending to a height of 1.3 m and presenting a shoulder width of 0.35 m, as visualized in Fig 2(a). The anatomical model of the infected patient was designed to continually emit viral particles from the mouth, which had dimensions of 0.05 m × 0.01 m. The mouth was situated approximately 0.15 m from the cranial apex, as delineated in Fig 2(b).

Mesh generation, a critical component of CFD simulation accuracy, was executed using ANSYS 2021. The mesh density was increased in areas where high velocity gradients were anticipated. Specifically, a minimum grid resolution of 0.002 m was allocated to the region around the patient’s mouth, while a slightly coarser grid with a resolution of 0.02 m was used to mesh the human figure and waiting- room windows. The clinic surface was meshed at a resolution of 0.05 m, whereas the broader zones of the computational domain were configured with a maximum grid size of 1 m. Structured grids were implemented across the model, and the total number of meshes was 4,823,377.

### 2.2 Physical modeling

The airflow dynamics within the established computational domain was comprehensively analyzed using a Eulerian frame. The continuous phase was resolved using time-averaged 3D Navier–Stokes equations for turbulent and transient flows, which serve as the governing equations for fluid motion. These equations, combined with the energy and continuity equations, provided a mathematical foundation for modeling the airflow and droplet dispersion in this study.

The Navier–Stokes equations are expressed as follows.

Continuity Equation:


$$\frac{\partial \rho }{\partial t}+\nabla \cdot (\rho u)=0.$$

Momentum Equation:


$$\frac{\partial (\rho u)}{\partial t}+\nabla \cdot (\rho uu)=-\nabla p+\nabla \cdot (\mu \nabla u)+F.$$

Here, ***ρ*** is the fluid density, ***u*** is the velocity vector, p is the pressure, μ is the dynamic viscosity, and ***F*** represents external forces such as gravity.

For the computational convergence of these fluid flow equations, including the turbulence equations, we adopted the SIMPLEC [38] (Jang et al., 1986) algorithm, which is a recognized and established scheme for fluid dynamics studies in such settings. Residuals for the primary flow equations (mass and momentum), as well as the turbulence equations governing turbulent kinetic energy (k) and its dissipation rate (ε), were meticulously monitored to ensure numerical convergence. These turbulence equations, central to the standard k–ε model, play a critical role in resolving the turbulence field and its interactions with the flow field. Convergence criteria were set to adequately capture the accuracy of the solution. The standard k − ε turbulence model was employed to address turbulence, which is fundamental to indoor airflow studies, particularly within confined spaces such as clinics. This model was considered appropriate for this study owing to its robustness and suitability for various flow scenarios. Standard wall functions were utilized to capture the near-wall flow physics more efficiently and ensure a more accurate representation of the flow characteristics close to the physical boundaries. This assumption is reasonable given the focus of the study on ventilation dynamics and is consistent with similar studies in the field. The ambient temperature was classified into two categories based on seasonal variations: T_cold_ (10 °C) and T_warm_ (30 °C), which represented winter and summer, respectively. Other critical air properties comprised a specific heat (Cp) of 1006 J/(kg·C), dynamic viscosity (μ) of 1.789 × 10^−5^ kg/(m·s), and thermal conductivity (k) of 0.0242 W/(m·C). Air was assumed to be dry in this simulation, implying that the potential effects of humidity variations were not incorporated.

The dispersion of droplets expelled from an infected individual is a pivotal aspect of this study. These droplets were diligently traced using discrete phase modeling. The trajectory of these droplets bearing the SARS-CoV-2 virus is governed by Newton’s second law, as represented by the following governing equation (Eq. 1) [39].


$$\begin{array}{c} m_{p}\frac{du_{p}}{dt}=F_{drag}+F_{gravitation}+F_{other} \end{array}$$ (1)

This equation encapsulates various forces acting on a particle, including drag, gravitational forces, and other forces such as the pressure gradient, Brownian forces, and rotational forces. The incorporation of the forces acting on airborne droplets is crucial for investigating their trajectory and dispersion within the established computational domain. The drag force $F_{drag}$ experienced by the droplets is expressed by Eq. (2), which involves two key parameters: the Stoke’s drag modification function $f_{D}$ and the characteristic response time of aerosol $\tau_{p}$.


$$F_{drag}=\frac{f_{D}}{\tau_{p}}\left(u-u_{d}\right),$$ (2)

where $f_{D}$ represents a corrective function that accounts for the effect of the Reynolds number on aerosol drag, particularly for larger droplets, wherein the inertial effects become substantial. $\tau_{p}$ indicates the time scale at which the particle adjusts to changes in the surrounding fluid phase.

The gravitational force $F_{gravitation}$ exerted on the droplets is described in Eq. (3).


$$F_{gravitation}=\frac{g\left(\rho_{d}-\rho \right)}{\rho_{d}},$$ (3)

where $\rho_{d}$ denotes the droplet density, which directly influences the gravitational descent of the droplets, consequently affecting their spatial distribution. Equations (4)–(6) were used to characterize $f_{D}$ and $\tau_{p}$ more meticulously. These equations incorporate parameters such as the droplet diameter $d_{p}$, turbulent viscosity $\mu_{t}$, and the molecular mean free path λ, all of which are fundamental in tailoring the calculations to emulate the actual droplet behavior in a turbulent transmission medium.


$$\begin{array}{c} f_{D}=1+0.15{Re}_{p}^{0.687} \end{array}$$ (4)

$$\tau_{p}=\frac{\rho_{d}{d_{p}}^{2}C_{c}}{18\mu_{t}}\;$$ (5)

$$\begin{array}{c} C_{c}=1+\frac{2\lambda }{d_{p}}\left[1.257+0.4e^{-\left(\frac{1.1d_{p}}{2\lambda }\right)}\right] \end{array}$$ (6)

The derivation of the forces and their associated parameters forms the foundation for simulations and analyses in this study, enabling an empirical approach for understanding the trajectory and dispersion of pathogen-laden droplets in clinical environments. A critical parameter in modeling droplet dynamics was the droplet density, which was set as ρ_drop_ = 1170 kg/m^3^, as proposed by Yang et al. [40]. The diameter of the droplets was set as 50 μm to ensure consistency with existing literature and simulation accuracy. These droplets were released in an unsteady state, and their flow rates and velocities were determined based on established research. The initial conditions play a pivotal role in ensuring realistic simulation outcomes. Key factors, such as exhalation temperatures for the summer and winter scenarios, were incorporated to reflect real-world conditions. Certain assumptions were made to simplify the simulations. First, the interaction between the droplets and airflow was neglected because of the low droplet concentration in the modeled environment, as supported by Yang et al. [40]. Second, the droplets were assumed to retain their spherical shapes throughout their trajectories. The exhalation process was idealized with a constant temperature and velocity, and the other occupants were assumed to inhale only, minimizing additional variables.

### 2.3 Simulation set-up

The computational domain was designed to replicate the realistic airflow conditions typical of a clinic waiting room. The inlet velocity was set at 5 m/s based on on-site airflow measurements in hospital waiting rooms. This value reflects the operational conditions of mechanical ventilation systems influenced by air-conditioning units, which are designed to rapidly dilute airborne contaminants and ensure effective air distribution in densely populated spaces. An outflow boundary condition was applied to the pressure outlet, which allowed unhindered airflow egress. The roof and lateral boundaries of the domain were modeled with symmetrical conditions reflecting the architectural layout of typical clinical spaces. To accurately simulate fluid-solid interactions, no-slip boundary conditions were applied to all walls, enabling the realistic representations of the airflow near solid surfaces. Human behavior was incorporated into the model, with the exhalation patterns of the infected individual simulated as steady and rhythmic, mimicking typical breathing styles. Droplet interactions with surfaces were modeled using trap conditions for seats, human body surfaces, and the floor, signifying that the droplets would be held at their point of contact. By contrast, an escape condition was applied to the inlet and outlet domains, allowing unrestrained droplet passage through these boundaries. ANSYS FLUENT 2021 was chosen for these numerical case studies because of its proven capability and reliability in fluid dynamics simulations. The transient nature of the flow demanded a time step Δt of 0.1 s, extending over a span of 120 s. Stringent convergence criteria were set to ensure the fidelity of the results. The scaled residuals for mass, momentum, turbulent, and species transport were constrained to values below 10^−4^. However, a stricter criterion was applied to the energy equation, necessitating residuals less than 10^−9^. These simulations were executed on a numerical intensive computing cluster using 32 processors. Notably, an approximate CPU time of 48 h was required to obtain a converged solution, underpinning the depth and complexity of the simulations. The monitoring of variables at the designated points is critical to ensure a stable evaluation process. Additionally, both the energy and mass balances were routinely evaluated, serving as indispensable metrics for determining convergence.

### 2.4 Validation of numerical model

Direct experimental validation was not feasible in this study because of the inherent challenges of replicating real-time aerosol dispersions under controlled conditions in clinic waiting rooms. However, the accuracy of the numerical model was indirectly validated by comparing the simulation results with findings from existing studies on airflow dynamics and droplet dispersion.

The predicted dispersion patterns of virus-laden droplets in this study aligned closely with the findings of previous studies. For instance, the observed buoyancy-driven vertical motion of droplets under warm conditions corresponded to the patterns reported by Wang and Hong [20] and Lipinski et al. [43], who highlighted the influence of temperature gradients on aerosol behavior. Similarly, the role of ventilation in mitigating droplet accumulation is consistent with experimental studies that emphasize the ability of airflow to dilute contaminant concentrations in confined spaces.

A mesh independence test was conducted to ensure that the results were not influenced by the grid resolution. Simulations were performed with progressively finer meshes, and the results showed negligible variation beyond a certain resolution, thereby confirming the stability and convergence of the numerical solutions.

Although experimental validation remains an ideal approach, these indirect validation steps provide a solid basis for confidence in the accuracy and applicability of the numerical models in real-world scenarios.

## 3 Results and discussion

Investigating the dispersion of virus-laden aerosols in clinic waiting rooms under various environmental conditions provides critical insights for designing effective preventive strategies for COVID-19. Six scenarios were simulated and categorized into hot and cold weather conditions, as presented in Table 1.

**Table 1:** Six working conditions employed in this CFD study.

Case	Ambient (°C)	Top ventilation	Windows
a	30	OFF	OFF
b	30	OFF	ON
c	30	ON	ON
d	10	OFF	OFF
e	10	OFF	ON
f	10	ON	ON

**Fig 3:**
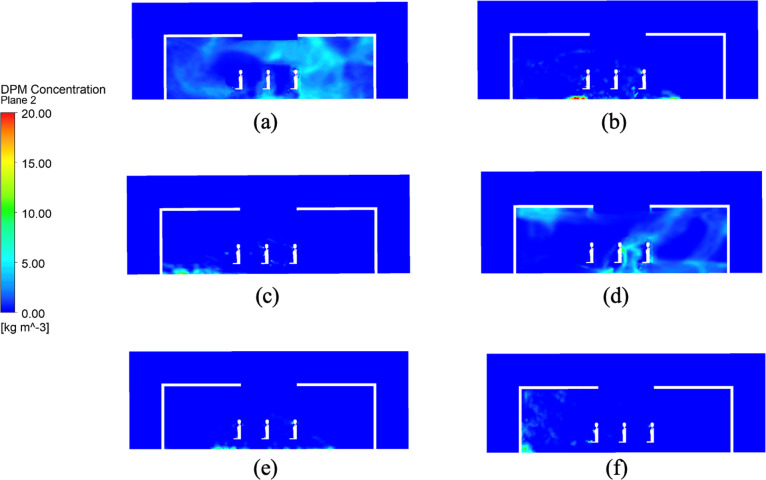
DPM concentration distribution on a vertical section at the room’s center for various working conditions.

**Fig 4:**
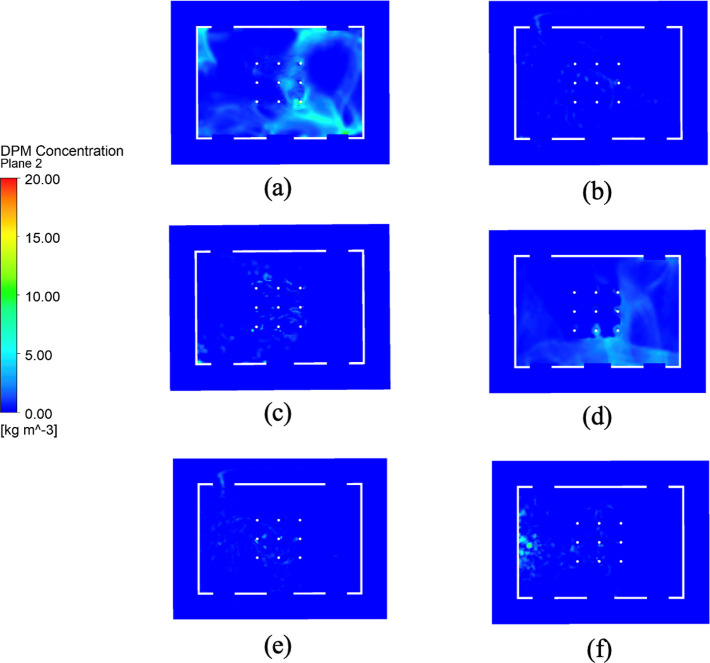
DPM concentration distribution on a horizontal section at human head height for different working scenarios.

Figs 3 and 4 illustrate the distribution of DPM concentration in the vertical section at the center of the room and in the horizontal section at the human head height, respectively. Significant variations are observed in virus-particle dispersion depending on the state of the ventilation device and windows. The average DPM concentration and total number of viral particles in each scenario were quantified, as presented in Table 2, which complements the above findings.

**Table 2:** Average DPM concentration (kg/m^3^) and number of virus particles under six distinct working conditions.

Case	Average concentration (kg/ m^3)	Number of particles (-)
a	5.80	238946
b	0.45	18469
c	0.18	7322d
d	4.75	195836
e	0.05	1866
f	0.04	1772

When both the top ventilation device and windows were closed (Scenarios a and d), the DPM concentrations were notably higher, indicating restricted airflow and viral entrapment within the room. Additionally, temporal analyses revealed that the peak viral concentrations occurred earlier under warmer conditions (Scenario a) than under colder conditions (Scenario d), suggesting that a stronger buoyancy-driven effect is observed in hot climates. For instance, at *t* = 50 s, the concentration at the head height in Scenario a was approximately 3.2 times higher than that in Scenario d. This observation supports previous studies emphasizing the importance of ventilation in reducing the amount of airborne contaminants [41,42].

Conversely, in scenarios where the windows were open, regardless of the state of the top ventilation device (Scenarios b, c, e, and f), the virus particle concentrations were considerably lower that observed when the windows were closed. Additionally, the viral concentration was predominantly localized near the floor. For instance, the lowest virus-particle concentration of 0.04 kg/m^3^ was observed in Scenario f (open windows and active top ventilation at 10 °C), which was 99.3% lower than that noted in Scenario a. This demonstrates that even minimal ventilation substantially mitigates the transmission of airborne viruses, aligning with the findings of Lipinski et al. [43], who advocated continuous ventilation to dilute indoor air pollutants.

A notable contrast was observed between the hot-weather Scenarios a and d, with the former exhibiting consistently higher DPM concentrations, particularly at the head height. This suggests that warmer temperatures enhance the persistence of airborne viral particles due to buoyancy effects, as corroborated by aerosol dynamics research [44]. Additionally, in Scenarios e and f (cold weather conditions), the virus concentrations were reduced to 0.05 and 0.04 kg/m^3^, respectively, further validating the effectiveness of combined ventilation strategies.

Additionally, the viral particle count in Scenario b was approximately 2.5 times higher than that in Scenario c, indicating that merely opening windows in warmer conditions is insufficient in reducing the viral count. Activating the top ventilation device was essential for effectively reducing the viral spread, underscoring the need for customized ventilation strategies based on seasonal and climatic variations. However, the difference in virus particle count was minimal between the cold-weather scenarios (Scenarios e and f), suggesting that open windows alone provided adequate ventilation in colder conditions, which is consistent with earlier studies [45].

**Fig 5:**
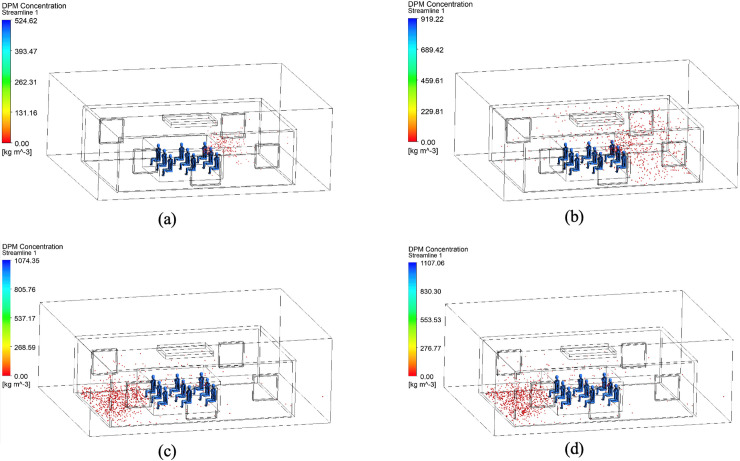
Temporal dynamics of viral distribution in a room under hot weather conditions with open windows and ventilation. (a) Virus accumulation at **t* *= 20 s around the carrier’s head; (b) Virus migration towards the rear at **t* *= 50 s; (c) Predominant virus concentration at the room’s front end at **t* *= 80 s; (d) Virus sedimentation towards the front floor area at **t* *= 120 s.

Fig 5 illustrates the temporal progression of viral dispersion in Scenario c, which represents a hot-weather scenario with both windows and top ventilation equipment open. At *t* = 20 s, the virus particles are concentrated primarily around the head of the carrier. At *t* = 50 s, the air currents direct the particles toward the rear seating and areas behind the carrier. Subsequently, the particles shift to the front by *t* = 80 s. In the final snapshot at **t* *= 120 s, the viral particles are significantly concentrated near the floor owing to the influence of gravity. This sequence highlights the combined effects of airflow dynamics and gravitational forces on aerosol dispersion, underscoring the critical role of optimized ventilation in reducing pathogen transmission in enclosed spaces.

The observed variations in DPM concentrations across different scenarios emphasize the complex interplay between airflow patterns and aerosol dynamics. Airflow characteristics, including direction, velocity, and turbulence, play a pivotal role in modulating viral dispersion, as demonstrated by numerous CFD studies on indoor air quality [46]. Gravitational sedimentation, prominently observed at *t* = 120 s, further underscores the importance of droplet size. Large droplets settle quickly owing to gravity, whereas small droplets remain airborne longer and can travel further under the influence of air currents [47]. Similarly, the findings of this study align with those reported by Sedighi et al. [48], who used CFD simulations of CO_2_ dispersion to assess population-level infection risk. Although Sedighi et al. focused on worst-case scenario distributions, their observed trends in pathogen dispersion and the critical role of ventilation are consistent with our results. These similarities further support the applicability of the CFD model employed in this study, particularly for evaluating the infection risks in enclosed indoor spaces.

Environmental factors not addressed in this study, such as humidity, warrant further investigation. Elevated humidity levels can promote droplet coalescence and alter the time viral particles remain airborne and sedimentation rates, as supported by previous research [49]. Room furnishings, which may serve as sinks or sources of viral particles through adsorption or resuspension, also contribute to the complexity of DPM dynamics in these environments [50].

### 3.1 Research limitations

Although this study provides valuable insights into aerosol behavior in clinic waiting rooms, it has some limitations. First, the computational model assumes steady environmental conditions, excluding sporadic variations, such as human movement or door openings, which are common in real-world settings. Second, this study focused on two specific temperature conditions that may not fully represent the diversity of global ambient environments. Third, humidity, a critical factor influencing aerosol dynamics, was not incorporated in this analysis as it directly affected droplet evaporation rates, altering their airborne lifetime and transmission distances. Under low-humidity conditions, rapid evaporation may reduce the droplet size to that of the nuclei, enabling the droplet to remain airborne for extended periods and travel further. Conversely, high humidity conditions slow down evaporation, leading to faster gravitational sedimentation. Neglecting this factor may lead to potential bias in the results, particularly when generalizing the findings to environments that exhibit considerable humidity variations. Surface contamination is another important factor that was not directly addressed in this study. Virus-laden droplets emitted by infected individuals may settle on chairs, tables, floors, or other surfaces, creating potential indirect transmission routes. This is especially critical in high-contact environments such as clinic waiting rooms, where individuals may unknowingly touch contaminated surfaces and subsequently touch their faces, thereby increasing the risk of infection. While our study primarily focused on airborne transmission, the role of surface interactions in overall transmission dynamics warrants further investigation. Future studies should integrate surface contamination with computational models to provide a more comprehensive understanding of COVID-19 transmission in enclosed spaces.

### 3.2 Future directions

Future studies can address these limitations by incorporating real-world variability such as transient human activities and dynamic ventilation adjustments. Exploring a wider range of ambient conditions, including diverse temperature and humidity levels, will enhance the generalizability of the findings. In particular, further investigations are required on the role of humidity, as it plays a crucial role in droplet evaporation, airborne persistence, and transmission distances. Further examinations into droplet-size distribution, surface interactions, and probabilistic modeling approaches can provide a more comprehensive understanding of aerosol behavior in indoor environments. Such research will be instrumental in refining infection control strategies for high-risk spaces such as clinic waiting rooms.

## 4 Conclusion

This study comprehensively investigated the dispersion dynamics of COVID-19 aerosols in clinic waiting rooms under various environmental and ventilation conditions using CFD. The results of this study underscore the critical role of effective ventilation systems, either through natural or mechanical means, in mitigating airborne transmission risks. Compared to closed environments, scenarios with open windows and active mechanical ventilation demonstrated significant reductions in virus-laden particle concentrations. These findings provide actionable insights for the design of safer healthcare spaces.

A key observation of this study was the enhanced persistence and dispersion of aerosols under warmer conditions owing to buoyancy effects, which amplify the risk of airborne transmission. This highlights the necessity for tailored ventilation strategies for different climatic conditions, particularly in warmer regions and seasons. This study also demonstrated how gravitational forces and airflow patterns influenced the spatial distribution and sedimentation of aerosols, emphasizing the importance of optimizing ventilation to minimize high-risk zones.

Furthermore, the novel incorporation of detailed human anatomical modeling and DPM technology enabled a more realistic simulation of droplet trajectories and aerosol behaviors in indoor environments. This methodological advancement bridges gaps in prior research by offering a more accurate representation of the dispersion of virus-laden particles and their interaction with environmental factors.

By presenting a detailed analysis of six distinct scenarios, this study provides evidence-based recommendations for healthcare facility design and operation. These findings advocate maintaining open windows and utilizing mechanical ventilation in high-risk spaces, such as clinic waiting rooms, especially under conditions that promote aerosol persistence. These insights have practical implications for improving infection control measures and reducing the risk of airborne transmission in enclosed, densely populated spaces.

In conclusion, this study advances our understanding of aerosol dynamics in healthcare settings and emphasizes the critical role of ventilation in reducing the airborne transmission risk. Although further research is required to incorporate real-world variability such as human movement and humidity effects, the findings presented herein offer a robust foundation for guiding infection control strategies and informing public health policies.
